# Emotional intelligence and recovering from induced negative emotional state

**DOI:** 10.3389/fpsyg.2015.00816

**Published:** 2015-06-19

**Authors:** Joaquín T. Limonero, Jordi Fernández-Castro, Jordi Soler-Oritja, María Álvarez-Moleiro

**Affiliations:** ^1^Research Group on Stress and Health, Facultat de Psicologia, Departament de Psicologia Bàsica, Evolutiva i de l’Educació, Universitat Autònoma de BarcelonaBarcelona, Spain; ^2^Facultat de Psicologia, Department of Clinical and Health Psychology, Universitat Autònoma de BarcelonaBarcelona, Spain

**Keywords:** emotional intelligence, emotional induction, IAPS, recovery, MSCEIT

## Abstract

The aim of the present study was to examine the relationship between emotional intelligence (EI) and recovering from negative emotions induction, using a performance test to measure EI. Sixty seven undergraduates participated in the procedure, which lasted 75 min and was divided into three stages. At Time 1, subjects answered the State-Trait Anxiety Inventory (STAI)-S, Profile of Mood States (POMS)-A, and EI was assessed by Mayer–Salovey–Caruso Emotional Intelligence Test (MSCEIT). At Time 2, negative emotions were induced by nine pictures taken from the International Affective Picture System and participants were asked to complete a second STAI-S and POMS-B questionnaires. At Time 3 participants were allowed to rest doing a distracting task and participants were asked to complete a third STAI-S and POMS-A questionnaires. Results showed that the branches of the MSCEIT emotional facilitation and emotional understanding are related to previous mood states and mood recovery, but not to mood reactivity. This finding contrasts nicely with studies on which emotional recovery was assessed in relation to EI self-reported measures, highlighting the perception and emotional regulation.

## Introduction

Over the last decades a substantial body of research has showed a positive association between emotional intelligence (EI) and adaptive use of emotions (Martins et al., 2010). EI has been defined as the ability of individuals to recognize, understand and regulate emotions, to discriminate among them, to use this information and to guide the thoughts and actions (Salovey and Mayer, 1990; Mayer and Salovey, 1997; Mayer et al., 2004). Mayer and Salovey (1997) model of emotional intelligence involves four branches, each of which represents a class of ability: (1) Perception of Emotions (2) Emotions to Facilitate Thinking; (3) Understanding and Analyzing Emotional Information and (4) Regulation of Emotion (Mayer and Salovey, 1997; Salovey et al., 2008).

Prior EI works were based in assessing this construct from self-reports (as e.g., Salovey et al., 1995, 2002; Fernández-Berrocal et al., 2004; Limonero et al., 2006b) or more extensive self-reported measures, including social processes and personality (i.e., Bar-On, 1997, 2000; Petrides et al., 2007; Siegling et al., 2015). Currently, most research measures EI through ability tests similar to those used in the measure of intelligence or cognitive performance. Out of these tests, one of the most used is the Mayer–Salovey–Caruso Emotional Intelligence Test (MSCEI; Mayer et al., 2002, 2003), developed by those who are considered the pioners of EI, term first used by Salovey and Mayer (1990). EI is one of the best predictors of adaptive coping strategies to stressful situations (Extremera and Fernández-Berrocal, 2002; Limonero et al., 2004, 2006a,b, 2012; Cabello et al., 2014). Limonero et al. (2006a) proposed that EI is a mediating variable between life events and their consequences on well-being. EI would facilitate appropriate responses to different events that a person would has to face daily and would decreases maladaptive emotional reactions by enhancing positive moods and reducing negative ones (Mayer and Salovey, 1997; MacCann et al., 2011). In other words, this set of abilities included in EI explains important personal life outcomes, and how a person differs from another one to face life events.

Furthermore, EI could be related to a simple emotional recovery, since a quick recovery from negative emotions would be very useful to cope faster in an adaptive way. Emotional recovery is the process of restoring equilibrium to the organism in terms of returning psychological and physiological activation to prior levels of an emotional reaction, especially if the emotional reaction comes from a negative external situation. Linden et al. (1997) observed that an impaired capacity of recovery from negative emotional states is more harmful to health than an acute rise of activation. Fredrickson (1998, 2001) and Fredrickson and Levenson (1998) have shown that recovery process from negative emotional states is not a passive change in terms of activation, but it is an active process that is promoted by positive emotions. Moreover, resilient individuals use positive emotions in order to recover from negative emotional states (Tugade and Fredrickson, 2004).

Only a handful of researches have related EI to emotional recovery (Salovey et al., 1995, 2002; Ciarrochi et al., 2001; Schutte et al., 2002; Petrides and Furnham, 2003; Fernández-Berrocal and Extremera, 2006; Arora et al., 2011). Overall, these studies indicate that individuals with high EI show lower negative mood states before and during emotion induction and in recovery, than lower EI individuals. In addition, Arora et al. (2011) concluded that individuals with high EI not only show a lower level of negative mood state in the recovery phase, but also a sharper recovery. However, it is worthy to note that in these studies the EI were based on self-reported measures resulting an important limitation because they did not really evaluate the EI of people since they are based on their self-perception. Given that, according to Brackett et al. (2006) and Joseph and Newman (2010) self-rated measures of EI may not be an accurate indicator of performance measures, and it may be valuable to check if relationship between emotional induction and recovery emotional and self-reported measures of EI, also occurs when the EI is measured by performance tests.

### Aim

The purpose of the present study was to examine the relationship between EI and recovering from negative emotions induction, measuring EI by a performance test. The first hypothesis was that individuals with higher EI will show lower negative states and higher positive states along a negative emotion induction process, including measurement at three time-points: previous to induction, during induction, and recovery after induction. The second hypothesis was that an interaction between EI and the process induction-recovery will arise; this means that people with high EI may react less on the induction phase and recover more in the third phase that people with low EI.

## Materials and Methods

### Participants

Undergraduate psychology students of the Autonomous University of Barcelona were asked to participate in a study on cognition and emotion; attendance was voluntary and participants received course credits to do so. Subjects signed also a consent form.

At first, 67 participants agreed, out of which three were excluded for not finishing any of the questionnaires administered. The final sample consisted of 64 participants. The average age was 22.32 (SD = 4.3). Most were female (78%).

### Assessment Instruments

Mayer–Salovey–Caruso Emotional Intelligence Test (MSCEIT version 2.0; Mayer et al., 2002). EI was measured by using a Spanish translation of the MSCEIT that shows similar psychometric properties to the original instrument (Extremera et al., 2006). The MSCEIT is a 141-item, ability-based measure with four branches of EI (perceiving, facilitating, understanding, and managing emotions) according to the theoretical model of Mayer and Salovey (1997). The instrument provides separate scores for each branch as well as an overall score for total EI. The scale had adequate reliability in this study (Cronbach’s alpha, 0.73). MSCEIT was scored using consensus criteria where each respondent’s answer is scored against the proportion of the sample that endorsed the same MSCEIT answer.

State-Trait Anxiety Inventory (STAI-S; Spielberger et al., 1970). The STAI is a 40-item questionnaire which provides separate measures of state and trait anxiety with 20 questions each. The Spanish validated form of STAI State was used (Buela-Casal et al., 2011) to measure subjective level of state anxiety experienced at the time of assessment. This 20-item questionnaire captures cognitive, emotional, and physical responses of anxiety. Participants rated each item on a 4-point scale (1: “*not at all*” to 4: “*very much*”), resulting in a minimum score of 0 and a maximum score of 60. Higher scores indicate greater levels of anxiety. The scales had adequate reliability (Cronbach’s alphas in the different time-points ranged between 0.92 to 0.94).

Profile of Mood States (POMS; McNair et al., 1971). The reduced Spanish validated form of POMS was used (Balaguer et al., 1993; Fuentes et al., 1995). This reduced version presents two equivalent forms: A and B. Each form has a 5-point scale (from 0: “*not at all*” to 4: “*extremely*”) of 15 items created to assess the following five affective mood states: Anger, Depression, Tension, Fatigue, and Vigor. The total mood disturbance score (TMDS) is obtained from scores of the other subscales. In this study the POMS presents an adequate reliability for all factors with Cronbach’s alphas ranging between 0.65 and 0.92, similar to the original scales (McNair et al., 1971). Participants completed three times the Spanish reduced version (A two times, and B one time).

### Procedure

#### Induction of Negative Emotional State

**FIGURE 1:**
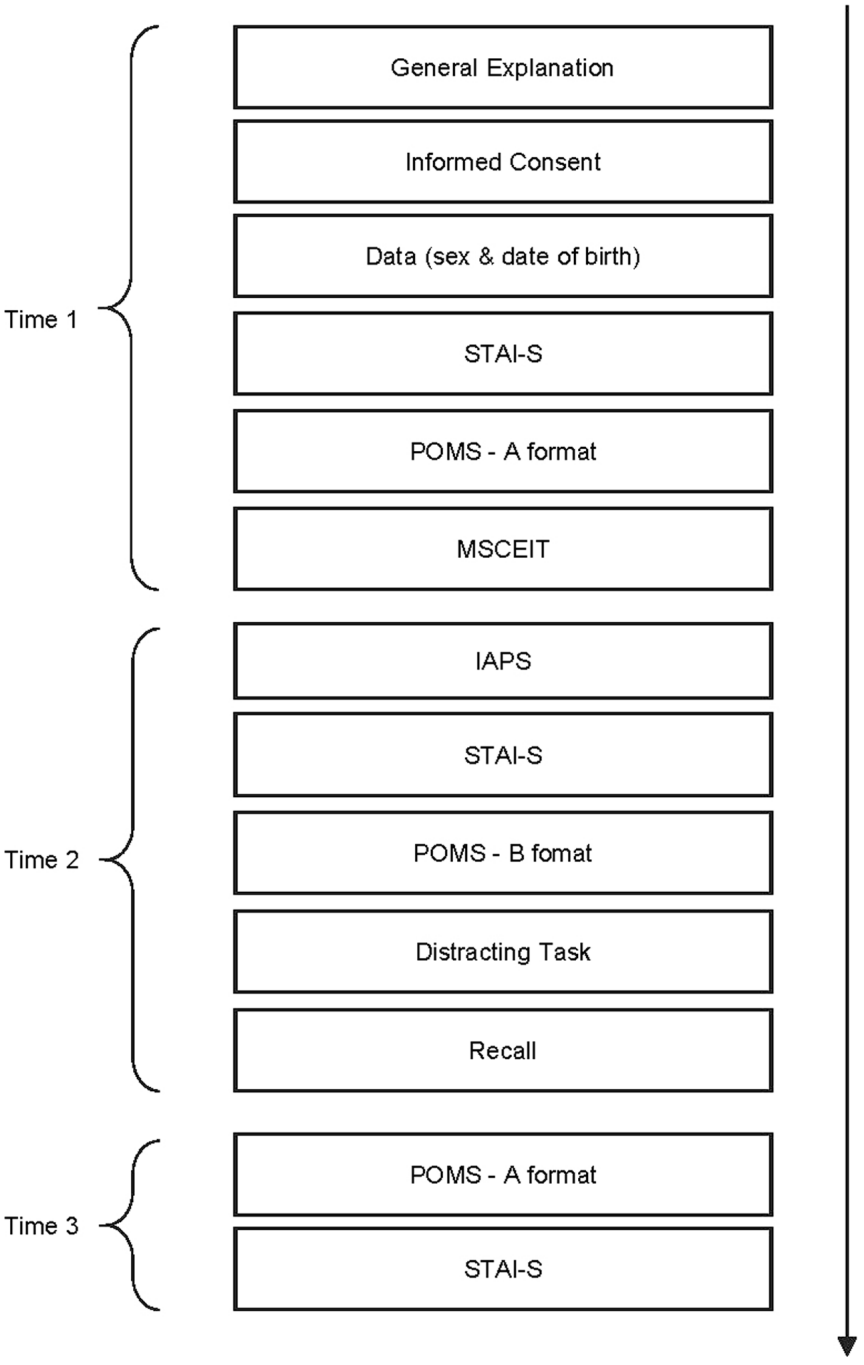
**General procedure**.

Participants individually arrived at the laboratory and were told that the study was designed to examine people’ emotional state and reactions to different situations. All participants voluntarily signed the written consent form after receiving a summary of the study. The procedure lasted 75 min and was divided into three stages (**Figure 1**). *Time1*. The subjects answered the different scales: STAI-S, POMS-A, MSCEIT, and also completed demographics variables (gender and age).

##### Time 2

For emotion induction, participants were individually shown nine pictures taken from the International Affective Picture System (IAPS; Lang et al., 1988, 2001). The 21-inch monitor was located at 1 m away from the participants, who were seated in a comfortable chair. The images were presented in the same sequence with an inter-trial interval of 18 s for all the participants. Images were chosen for negative valence and high activation (**Table 1**) in accordance with the Spanish validation norms of the IAPS (Moltó et al., 1999; Vila et al., 2001). Before passing out these pictures, three images were used as examples to explain subjects how to assess throughout the *Self-Assessment Manikin* (SAM; Lang, 1980). SAM is a non-verbal pictorial affective rating system that uses graphic figures to depict values along the dimensions of activation and valence in a 9-point rating scale ranging from 1 (the lowest rating) to 9 (the highest rating). These images were different from those used in emotional induction. The images chosen in the present study present medium high activation (*M* = 7.32, SD = 1.96) and negative valence (*M* = 1.69, SD = 1.13) measured by SAM and their reference identifications are indicated in **Table 1**. After participants viewed the pictures from IAPS they completed a second STAI-S and POMS in form B. Then participants were allowed to rest in the same laboratory room for 15 min doing a distracting task, which involved answering the following five questions: “*What are your favorite colors?*” “*What are your favorite songs*?” “*What are your favorite movies*?” “*Describe three important memories of your life*,” and “*What are your favorite flavors*?

**Table 1:** Valence and Arousal Values of the IAPS for the selected images of the study.

Presentation order		Valence		Arousal	
Reference	Description	*M*	SD	*M*	SD
2683	War	1.68	1.14	7.55	1.74
1525	Attack dog	2.72	1.64	7.79	1.61
6315	Beaten fem	1.81	1.23	7.55	1.77
9635.1	Man on fire	1.77	1.28	7.45	1.84
9433	Dead man	1.55	1.29	6.88	2.45
3015	Accident	1.46	1.14	7.48	1.9
2095	Toddler	1.23	0.67	7.19	2.13
3301	Injured child	1.54	0.94	7.05	2.04
9265	Hung man	1.45	0.85	6.94	2.19
	**Mean** (SD)	**1.69**	(1.13)	**7.32**	(1.96)

*IAPS, International Affective Picture System; IAPS Spanish validation norms (Moltó et al., 1999; Vila et al., 2001)*.

##### Time 3

After the 15 min break, participants were asked to complete a third STAI-S and POMS-A form. Finally, the researchers debriefed participants.

### Statistical Analysis

Data were analyzed using the Statistical Package for Social Sciences (Version 20.0; IBM, USA). The internal consistency reliability of the different scales was evaluated by Cronbach’s alpha coefficient. Comparison of means and repeated measures General Linear Models (GLM) with two between-subject factors were used to test the hypotheses. The extreme groups in the different branches of EI (Perception, Facilitation, Understanding, and Managing) were formed with the quartile 1 and 3 criteria. Results were considered statistically significant at *p* < 0.05. Bonferroni correction was used to adjust significance levels for multiple pair comparisons.

## Results

There were no significant differences between male and female students on the variables of the study (**Table 2**).

**Table 2:** Descriptive statistics of the different variables of the scales and comparison between sex.

Variables	*M* (SD)	CI 95%	*p∗*
MSCEIT Branch 1 Perceiving	0.48 (0.093)	(0.46, 0.51)	0.615
MSCEIT Branch 2 Facilitating	0.41 (.059)	(0.40, 0.43)	0.408
MSCEIT Branch 3 Understanding	0.49 (0.052)	(0.47, 0.50)	0.745
MSCEIT Branch 4 Managing	0.39 (0.047)	(0.38, 0.41)	0.626
MSCEIT Experiential Area	0.45 (0.069)	(0.43, 0.46)	0.516
MSCEIT Strategic Area	0.44 (0.035)	(0.43, 0.45)	0.935
Overall MSCEIT	0.44 (0.044)	(0.43, 0.45)	0.721
STAI-S Pre-IAPS	15.16 (8.592)	(13.01, 17.3)	0.531
STAI-S Post-IAPS	25.91 (11.790)	(22.96, 28.85)	0.531
STAI-S Follow-up	19.64 (11.773)	(16.7, 22.58)	0.102
POMS Overall Pre-IAPS	107.34 (9.058)	(105.08, 109.61)	0.495
POMS Tension Pre-IAPS	4.91 (2.659)	(4.24, 5.57)	0.694
POMS Depression Pre-IAPS	2.20 (2.154)	(1.67, 2.74)	0.384
POMS Anger Pre-IAPS	2.70 (2.355)	(2.11, 3.29)	0.824
POMS Fatigue Pre-IAPS	4.64 (2.698)	(3.97, 5.31)	0.781
POMS Vigor Pre-IAPS	7.11 (2.761)	(6.42, 7.8)	0.119
POMS Overall Post-IAPS	110.58 (9.498)	(108.21, 112.95)	0.217
POMS Tension Post-IAPS	5.94 (3.221)	(5.13, 6.74)	0.183
POMS Depression Post-IAPS	2.05 (2.264)	(1.48, 2.61)	0.309
POMS Anger Post-IAPS	3.86 (3.241)	(3.05, 4.67)	0.838
POMS Fatigue Post-IAPS	3.73 (2.632)	(3.08, 4.39)	0.600
POMS Vigor Post-IAPS	5.00 (2.417)	(4.4, 5.6)	0.217
POMS Overall Follow-up	99.84 (8.175)	(97.8, 101.89)	0.134
POMS Tension Follow-up	2.41 (2.926)	(1.68, 3.14)	0.170
POMS Depression Follow-up	0.86 (1.542)	(0.47, 1.24)	0.179
POMS Anger Follow-up	0.91 (2.187)	(0.36, 1.45)	0.643
POMS Fatigue Follow-up	2.39 (1.857)	(1.93, 2.85)	0.527
POMS Vigor Follow-up	6.72 (2.367)	(6.13, 7.31)	0.113
IAPS Mean Valence	1.66 (.609)	(1.51, 1.81)	0.281
IAPS Mean Arousal	7.19 (1.131)	(6.9, 7.47)	0.056

*MSCEIT, Mayer–Salovey–Caruso Emotional Intelligence Test; STAI-S, State-Trait Anxiety Inventory (State); POMS, Profile of Mood States; IAPS, International Affective Picture System. ∗p = value of significance related to Mann–Whitney U test*.

As shown in **Table 3**, mood induction conditions had a powerful impact on STAI State and on POMS Total and on each of their components. Mood induction had elicited more negative than positive sentences in recovery phase (*t* = 9.28, *df* = 63; *p* < 0.001). This result indicates that the experimental manipulation had the desired effect.

**Table 3:** Mean of STAI-S and POMS Total and each of their components in each time-point.

	Time 1	Time 2	Time 3
	Baseline	Post emotional induction	Recovery
	*M* (SD)	*M* (SD)	*M* (SD)
STAI-S Anxiety	15.16 (8.592)	25.91 (11.790)^a∗∗^	19.64 (11.773)^b∗∗^
POMS Total	107.34 (9.058)	110.58 (9.498)^a∗^	99.84 (8.175)^b∗∗^
POMS Tension	4.91 (2.659)	5.94 (3.221)^a∗^	2.41 (2.926)^b∗∗^
POMS Depression	2.20 (2.154)	2.05 (2.264)^a∗^	0.86 (1.542)^b∗∗^
POMS Anger	2.70 (2.355)	3.86 (3.241)^a∗^	0.91 (2.187)^b∗∗^
POMS Fatigue	4.64 (2.698)	3.73 (2.632)^a∗^	2.39 (1.857)^b∗∗^
POMS Vigor	7.11 (2.761)	5.00 (2.417)^a∗∗^	6.72 (2.370)^b∗∗^

*STAI-S, State-Trait Anxiety Inventory (State); POMS, Profile of Mood States*.

*M, Mean; SD, Standard Deviation*.

*^a^comparison time 1 vs. time 2*.

*^b^comparison times 2 vs. time 3*.

*∗p < 0.05; ∗∗p < 0.001*.

The hypotheses of this study were tested separately for each of the branches of the IE way, so results will be exposed for each of them.

### EI Perception

A GLM of the effects of negative emotional induction on STAI-S showed that there was a significant effect of mood induction conditions (*F* = 19.721, *p* < 0.001). *Post hoc* analysis revealed that STAI-S score at time 3 (recovery) was higher than score at Time 1 (*F* = 13.070, *p* < 0.001) and lower than score at Time 2 (*F* = 8.533, *p* < 0.01). The difference between pre induction (Time 1) and mood induction (Time 2) was significant (*F* = 45.87, *p* < 0.001). A slightly main effect of EI-Perception was found (*F* = 3.797, *p* = 0.061), and there was no interaction between factors.

A GLM of the effects of negative emotional induction on the total score of POMS also showed a significant effect of mood induction conditions (*F* = 20.29, *p* < 0.001), with statistically differs along the three time-points. However, there was no effect related to Emotional Perception. Then, there were performed two different GLM: the first one of the Vigor score of POMS and the other one for the Tension score. The GLM of Vigor showed none main effect due to Emotional Perception, however, the GLM of Tension showed a slightly main effect of Emotional Perception (*F* = 3.298, *p* = 0.070) and there was not interaction between factors.

In summary, for the perception branch of EI, neither of the two hypotheses of the study has been confirmed. However, people with higher values in Emotional Perception showed less recall of negative phrases (*U* = 72, *p* < 0.05) and more of positive ones (*U* = 184, *p* < 0.05) in the recuperation phase.

### EI Facilitation

**FIGURE 2:**
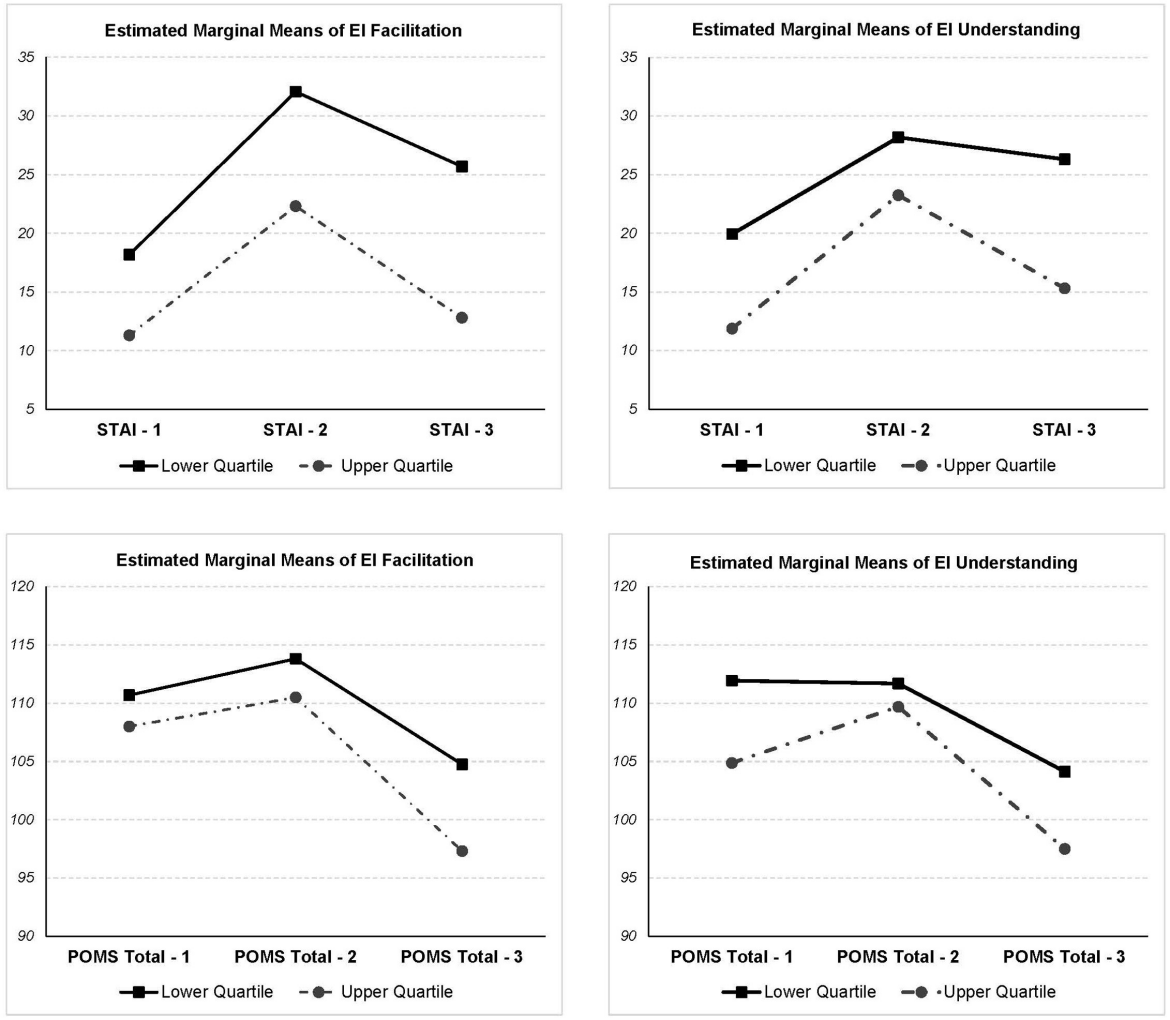
**Comparison of estimated marginal means of Emotional Facilitation and Emotional Understanding branches of MSCEIT**. Observation: STAI-S = State-Trait Anxiety Inventory (State); POMS = Profile of Mood States.

The GLM of the effects on STAI-S showed that there was a significant effect of mood induction conditions (*F* = 22.063, *p* < 0.001). *Post hoc* analysis revealed that STAI-S score at Time 3 (recovery) was higher than score at Time 1 (*F* = 8.609, *p* < 0.01) and lower than score at Time 2 (*F* = 17.482, *p* < 0.001). A main effect of Emotional Facilitation was found (*F* = 13.767, *p* < 0.001), and there was no interaction between factors (see **Figure 2**, upper left frame).

The GLM of the effects on total score of POMS showed a significant effect of mood induction conditions (*F* = 16.887, *p* < 0.001), with statistical differences along the three times. There was a main effect of EI Facilitation (*F* = 4.374, *p* < 0.05) and there was no interaction between factors (see **Figure 2**, lower left frame).

The GLM of Vigor showed no main effect due to Emotional Facilitation, however, the GLM of Tension showed a main effect of Emotional Facilitation (*F* = 10.183, *p* < 0.01) and there was an interaction between Tension scores and EI Facilitation (*F* = 4.835, *p* < 0.001). *Post hoc* contrasts revealed that the group with higher EI Facilitation showed a decrease between Time 1 and Time 3 steeper than the lower EI Facilitation group (*F* = 8.388, *p* < 0.01). In short, the first hypothesis was confirmed: individuals with higher facilitation branch of EI showed lower levels of anxiety along the three times of measurement. Regarding the second hypothesis, which refers to the interaction between EI and recovery, is only true in relation to the negative mood states, being individuals with more facilitation which decrease more the negative mood states during recovery. Furthermore, in the Recovery phase, people with higher values in Emotional Facilitation showed less recall of negative phrases (*U* = 76, *p* < 0.05) and more of positive ones (*U* = 180, *p* < 0.05).

### EI Understanding

The GLM of the effects on STAI-S showed that there was a significant effect of mood induction conditions (*F* = 14.181, *p* < 0.001). *Post hoc* analysis revealed that STAI-S score at Time 3 (recovery) was higher than score at Time 1 (*F* = 7.829, *p* < 0.01) and lower than score at Time 2 (*F* = 7.193, *p* < 0.01). A main effect of Emotional Understanding was found (*F* = 5.113, *p* < 0.05), and there was no interaction between factors (see **Figure 2**, upper right frame).

The GLM of the effects on the total score of POMS showed a significant effect of mood induction conditions (*F* = 19.353, *p* < 0.001), with statistical differences along the three times. There was a main effect of Emotional Understanding (*F* = 5.113, *p* < 0.05) and there was no interaction between factors (see **Figure 2**, lower right frame).

The GLM of Vigor showed no main effect due to Emotional Understanding, however, the GLM of tension showed a main effect of Emotional Understanding (*F* = 5.735, *p* < 0.05) and there was no interaction between factors. In short, individuals with higher understanding branch of EI show lower levels of anxiety and negative mood states along the three times of measurement, so the first hypothesis was fulfilled. But the second hypothesis related to interaction was not confirmed. Furthermore, no difference was observed between the high and low levels of Emotional Understanding and the number of positive (*U* = 136, *p* > 0.05) and negative sentences (*U* = 120, *p* > 0.05) recalled.

### EI Management

There were performed GLM of STAI and POMS with the comparison between the group of higher EI Management and the group of lower Emotional Management. No effects were found when Vigor scores or tension scores of POMS were analyzed, either. So, for the perception branch of EI, neither of the two hypotheses of the study has been confirmed. Furthermore, no difference were observed between the high and low levels of Emotional Management and the number of positive (*U* = 155.5, *p* > 0.05) and negative sentences (*U* = 105.5, *p* > 0.05) recalled.

## Discussion

The induction of negative mood has been effective since subjects showed more anxiety, more negative moods (POMS total) and less vigor during the induction. Moreover, recovery after induction took place. Also we have observed that mood induction elicited more negative than positive sentences demonstrating the goodness of mood induction through selected IAPS pictures as observed in others studies (Mikels et al., 2005; Feliu-Soler et al., 2013).

Overall, results suggest that the branches of the MSCEIT Emotional Facilitation and Emotional Understanding are aspects of EI related to previous mood states and mood recovery, but not to mood reactivity. Emotional Facilitation has a main effect on anxiety and mood state. This means that emotional intelligence has two influences: first the negative emotional levels are lower in individuals with higher EI along the three phases of the process, and second, these individuals shown high recuperation. The analysis of the components of POMS shows that the influence of EI is mainly dropping down negative mood states and rising up the positive ones. This means that it has been found an interaction effect in individuals with higher EI recover n more than individuals with lower EI.

People with higher levels of Emotional Facilitation showed a more positive mood state. This state influences what people think and plays an important role in the recovering process toward generating positive thoughts (Mayer and Salovey, 1997), in our case, positive memories. In line with this, Baikie and Wilhelm (2005) have observed that writing about a meaningful aspect of life when one person is under negative emotional state may result in enhanced positive emotions and reduce the effect of negative ones. On the other hand, the perception branch of EI also produces positive memories during recovering, but has neither effect on the negative mood states nor in the anxiety level. This incongruous fact should be clarified in future studies.

Emotional Understanding influences also recovery from negative emotional states. Emotional Understanding influences recovery in the same way as facilitation does, but no interactive effect has been found. In this sense, what gives rise to different emotions is a key component of EI. This fact includes the ability to understand emotional information, the manner in which they combine, and their causes and consequences. In the above-cited study of Fernández-Berrocal and Extremera (2006), the authors found that Emotional Perception (dimension of perceived emotional intelligence measured by Trait Meta-Mood Scale, Salovey et al., 1995) moderated mood reactivity, diminishing intrusive thoughts and negative mood. In another natural setting, Limonero et al. (2004) found that a higher level of Emotional Clarity reduces stress levels of nurses to facilitate the understanding of personal relationships, professional and intragroup communication. Ruiz-Aranda et al. (2014) found that among female student health professionals global EI measured by MSCEIT was an important predictor of well-being to assess situations as less stressful.

Emotional Perception has a weak effect on emotional induction and recovery. In fact this is not a surprise because the perception doesn’t need to be related to the intensity and the change of emotional states, but with emotional states identification. In this point, it is important to note that perception has been measured by performance test. To believe that oneself has a good perception of his emotions it is not the same as to prove this skill for real. This effect has been observed in other applications of EI. For instance, empirical studies on burnout show that while perceived EI has been clearly related to burnout, even controlling for personality traits (Mikolajczak et al., 2007), the results are mixed in terms of the relationships between EI as a skill and emotional exhaustion, either no relationships were found (Brackett et al., 2010) or relations were only found with some components of EI (Palser, unpublished doctoral dissertation).

Emotional Management has not showed influence on recovery from mood induction. This result was not expected because people with higher Emotional Management or emotion regulation should show more emotional recovery to experience less stress (Limonero et al., 2004; Ruiz-Aranda et al., 2014; Peña-Sarrionandia et al., 2015). One possible explanation to these unexpected results could be related to the experimental procedure: subjects experienced negative emotions from mood induction procedure, and they did not face any active task to cope with negative moods afterward, so they might not mobilized active resources related to management emotion as for example, repairing or reducing the negative emotions.

The results provide mixed support for the initial hypothesis; only the Emotional Facilitation and Emotional Understanding components of EI were related to the recovery process after mood induction. In relation to the second hypothesis, we note that it was also partially confirmed.

In summary, in this study it has been found that Emotional Understating and Emotional Facilitation assessed by an ability-based measure are the key branches in promoting recovery in negative emotions induction settings, while EI is evaluated by self-report measures the main branches are perception and management. This result invited to reflect and draw practical conclusions: people believe that perceiving and controlling emotions help to recover better from negative emotional states, but in fact, according to the presented results, people who do use emotions to guide cognitive process are those who recover better. This suggests that recovery does not depend on controlling emotions, but it is related to more undirected processes as Fredrickson (1998, 2001) claims. His model promotes positive things to restore equilibrium. Therefore training for managing stressful situations would be based on facilitation of resources more than on direct control activities. Having said this, it would be useful to promote emotional capacities related to the components of emotional facilitation and understanding to cope more effectively with negative situations.

These findings are particularly applicable to situations of communicating bad news as serious diagnoses in a clinical setting, since it is a passive situation of receiving threat information. In this case, emotional facilitation and understanding could reduce the levels of negative emotions and thereby improve patient’s understanding and retention of information. It also facilitates the involvement in their treatment plan and in the different ways of coping with a diagnosis of illness or with the illness (Edo et al., 2012).

### Limitations and Future Research

Despite the insights that the present study provides, it does have several limitations. Firstly, a high percentage of the participants were female, so there is a possibility that findings may not be generalized to males. Secondly, participants were university students, so we must be cautious about generalizing the results to the general population. Future studies should increase the sample and include general population and increase the proportion of men to analyze data for possible gender differences. Another limitation of this research is that it has only been induced one kind of negative emotion, anxiety. In future research the induction and recovery of anxiety should be compared with other emotions as sadness and/or anger and it would also be interesting to identify the resources and strategies that emotionally intelligent people use to reduce negative effect.

Regardless of these limitations, the present study suggests that Emotional Facilitation and Emotional Understanding branches of EI are related to previous mood states and with mood recovery from negative mood induction.

## Conflict of Interest Statement

The authors declare that the research was conducted in the absence of any commercial or financial relationships that could be construed as a potential conflict of interest.
